# Pre-market health systems barriers and enablers to infectious diseases point-of-care diagnostics in Australia: qualitative interviews with key informants

**DOI:** 10.1186/s12879-024-10214-5

**Published:** 2024-11-19

**Authors:** Lise Lafferty, Tanya L. Applegate, Sophie Lewis, Kerryn Drysdale, Robert Monaghan, Angela Kelly-Hanku, Rebecca Guy, Carla Treloar

**Affiliations:** 1 Centre for Social Research in Health, UNSW Sydney, Sydney, Australia; 2 The Kirby Institute, UNSW Sydney, Sydney, Australia; 3 Sydney School of Health Sciences, University of Sydney, Sydney, Australia

**Keywords:** Infectious diseases, Point-of-care testing, Qualitative research, Regulatory frameworks, Sustainability

## Abstract

**Background:**

There exist multiple regulatory layers for point-of-care (POC) testing to be implemented within Australia. This qualitative analysis sought to understand the pre-market barriers and facilitators to scale-up infectious diseases POC testing in primary care settings at the national level.

**Methods:**

Key informant interviews were undertaken with people (*n* = 30) working in high- level positions relevant to infectious diseases POC testing in Australia. Participants were recruited from federal and state health departments, industry, and nongovernment national peak bodies. The Unitaid scalability framework informed this analysis to understand barriers and enablers to creating access conditions and establishing country readiness for market access of POC tests.

**Results:**

Participants identified regulatory frameworks as significant barriers to market access. National strategies and advocacy were viewed as potential enablers to establishing country readiness. It was recommended that the national system for universal health care should fund infectious disease POC tests to ensure financial sustainability, though the existing pathology infrastructure was regarded as a likely inhibitor.

**Conclusions:**

Current regulatory frameworks inhibit market access for infectious disease POC testing devices for use in the primary care setting. National advocacy is urgently needed to gain government support and align national policies with regulatory frameworks.

**Supplementary Information:**

The online version contains supplementary material available at 10.1186/s12879-024-10214-5.

## Introduction

Point-of-care (POC) testing is a laboratory test occurring at, or near, the patient which provides a rapid result to facilitate improved clinical care (such as prompt treatment access) [1]. Internationally, POC testing has been used in a range of decentralised settings such as district hospitals, primary care clinics or the community, where there is either no access to pathology laboratories or access may be impeded or slow [2], including in remote and resource-limited settings [3, 4].

Integration of diagnostic tools within the primary care setting can improve patient safety in primary health care by reducing diagnostic errors, enabling accurate prescribing, that in turn reduces the antimicrobial resistance caused by the unnecessary use of antibiotics [5]. POC tests for infectious diseases have primarily been adopted in settings with limited laboratory infrastructure [6]. The main decentralised programs in Australia have been government funded demonstration projects focused on key populations such as sexually transmissible infection testing in remote Aboriginal communities [7, 8], which was extended to COVID-19 [9]. Hepatitis C virus POC testing has been implemented as part of a national program to reach people at higher risk of hepatitis C virus exposure, including people who inject drugs [10].

POC infectious disease testing can facilitate engagement in care for people who may not regularly access healthcare, such as hepatitis C testing among people who inject drugs [11, 12]. Molecular POC testing for sexually transmissible infections and COVID-19 have been successfully scaled up in rural and remote primary care settings in Australia [9, 13]. While POC testing scale up can be achieved, a review published in 2020 found several challenges which inhibit implementation of POC testing in resource-limited settings despite availability of these technologies [14]. Such bottlenecks to scale up included navigable regulations, political will, funding, and workforce retention and training [14]. However, few research studies have examined broader health systems barriers and enablers for national scale-up of POC testing for infectious diseases (see for example: [15]), such as consideration of regulatory frameworks which foster or inhibit market access prior to implementation. While these new technologies have the capacity to transform clinical care, through bringing diagnostics to the patient, there is limited information about what is needed at the health systems level to ensure pre-market country readiness for POC diagnostics.

The regulatory ecosystem within Australia encompasses market access via assessment from the Therapeutic Goods Administration (TGA). The cost for application to the TGA (the government stringent regulatory authority responsible for evaluating, assessing and monitoring therapeutic goods) is a listed fee and not proportionate to disease prevalence [16]. When assessing potential interventions for the Australian market, the TGA applies a risk classification framework to determine “the amount of scrutiny” applied to assessing medical devices, with greater evidence required for higher risk classifications [16]. Specifically, in vitro diagnostics (IVDs) (i.e., tests which detect disease which pose “a high public health risk due to the significant impact incorrect results would have for public health” fall under rule 1 of the classification of IVD medical devices, and are consequently classified to the highest level (Class 4) ([17]:8). Australia’s universal healthcare is delivered under Medicare, with many health services subsidised under the Medicare Benefits Schedule (MBS) [18]. For health interventions to be eligible for reimbursement under the MBS or other Medicare scheme, an application must be submitted to the Medical Services Advisory Committee – an independent non-statutory committee responsible for appraising newly proposed pathology and clinical services and advising the Australian Government prior to listing on the MBS [19]. Of note, subsidies are provided to public patients through Medicare for services such as consultations with general practitioners, with many pathology tests available (via general practitioner referral) at no cost to the patient [20].

The Unitaid scalability framework is a guide for global and national level scale-up of healthcare interventions [21]. Specifically, the framework focuses on three key considerations for scale up: (1) creating global conditions for scale-up, including sustainable access conditions, alignment and coordination with global donors and partners, and generation and dissemination of knowledge and evidence; (2) establishment of country readiness for scale-up, including securing political and financial support, ensuring programmatic and operational readiness (i.e., implementation preparedness), and creating community-driven demand; and (3) transition, e.g., transition of funding and other resources to services to enable and support scale-up (regarded as a step towards scale-up though not necessarily the end goal); with these combined efforts culminating in country-level commitments for scale-up [21]. This scalability framework focuses on broader considerations for national scale-up at a health systems level and was thus selected as the framework to inform this analysis. This qualitative analysis utilises the Unitaid scalability framework to: (1) understand the barriers and enablers to creating national conditions for scale up; and (2) identifying country readiness for sustainably scaling up an infectious diseases POC testing model in the primary care setting in Australia.

Key stakeholders who may be involved in or might influence POC testing scale-up were engaged to provide their expert insights.

## Methods

This qualitative study was conducted within the National Health & Medical Research Council Centre of Research Excellence-funded RAPID Point-of-care research consortium for infectious disease in the Asia Pacific and draws on data from semi-structured interviews with people working in high-level positions relevant to infectious diseases POC testing in Australia. A list of potential interview participants – i.e., people in leadership positions in national or jurisdictional departments/organisations who would be likely to be involved in providing advice on scale-up decisions – was developed in consultation with Chief Investigators of the RAPID investigator group. Potential participants were nominated based on their expertise and/or professional leadership in one or more of the following areas relevant to infectious diseases POC testing and scale-up: infectious disease (including specific infectious diseases, e.g., HIV, STIs, viral hepatitis, COVID-19), population specific focuses (e.g., prisoner health, Aboriginal and Torres Strait Islander health, gay men’s health), point-of-care manufacture, accreditation, quality assurance, pathology / laboratory, health economics, and workforce training. The names of potential participants were compiled into a spreadsheet tabulated by expertise and professional affiliation. To ensure timely scheduling of interviews, a small number of invitations were sent at a time to people working in diverse areas, ensuring broad representation of expertise throughout data collection. Potential interviewees were invited to participate via email, either from the interviewer or from the Chief Investigator who nominated them. Follow up correspondence occurred only between participant and interviewer to ensure confidentiality of those who accepted/declined participation. A total of 60 people were invited to participate, of which 30 were interviewed. Twenty-two of those invited did not reply to invitations, five could not commit time to the interview, two perceived they did not have the relevant content knowledge, and one person did not provide a reason.

The interview guide (see Supplementary File 1) was designed to understand participants’ perceptions of enablers and barriers to scale up of infectious diseases POC diagnostics, including market entry and regulatory frameworks, identification of key stakeholders, definitions of POC testing and scale-up, perceptions of knowledge/evidence relied upon when making decisions about scale-up, health system enablers and barriers to scale-up, and challenges for implementation. Two pilot interviews were conducted by two authors to ensure questions were relevant and applicable; the interview guide was adjusted accordingly. Interviews were conducted over video conference or phone, between July 2021 and February 2022, and typically lasted 50 min. The study team met fortnightly throughout data collection and discussed interview content and participant perspectives.

Interviews were conducted by two authors (LL and SL), both of whom are experienced qualitative researchers. To ensure participant anonymity, the names of those who agreed/declined participation were not shared with the RAPID investigator team. The Participant Information Statement was attached to the initial email invitation and reviewed with participants prior to interview commencement. Informed verbal consent was obtained from all participants prior to commencement of interviews; participants were not remunerated. The researchers decided that the data collected during the thirty interviews were sufficient to provide answers to the study research questions.

Interviews were audio-recorded then transcribed and proofread for accuracy. De-identified transcripts were uploaded to NVivo (version 12) qualitative software. The coding framework was developed among the study team and informed by scale-up and implementation frameworks. Two frameworks were considered to guide analysis, including a patient-centred model specific to scaling up POC testing [9], and a scale up guide for population health interventions [22]. This analysis focuses on the pre-market considerations of POC testing, specifically regulatory frameworks and identification of key stakeholders. Given the health systems focus of this analysis, the Unitaid scalability framework was retrospectively applied [21]. Deductive coding was informed by the abovementioned frameworks, with an additional node allocated for ‘barriers and enablers’. The barriers and enablers were then cross-referenced with the other various nodes to ensure all relevant data was captured. All barriers and enablers relating to pre-market concerns (e.g., regulatory frameworks, sustainable funding, market access, creating government buy-in, advocacy) were then thematically organised according to the Unitaid scalability framework.

Ethics approvals were obtained from: University of New South Wales Human Research Ethics Committee (HC200472), and Australian Institute of Aboriginal and Torres Strait Islander Studies Research Ethics Committee (EO209-20201002).

All participants whose quotes were selected for use within this manuscript were given the opportunity to approve/deny inclusion of excerpts via email (none rejected). Quotes are presented with participant number only to ensure anonymity.

## Results

Thirty people, including 12 women and 18 men, participated in a semi-structured interview. All but two interviews were conducted one-on-one, with the remaining two having two participants each. Participant positions included CEOs, directors (and comparable positions), professors, and chief medical officers, with six participants holding mid-level positions being nominated for their content expertise.

Focusing on Unitaid’s ‘conditions for scale-up’ and ‘country readiness’, barriers and enablers were identified across these domains, specifically, the role of regulatory frameworks as part of ‘sustainable access conditions’ (conditions for scale-up), and ‘establishing country readiness for scale-up’, including supportive policies, political engagement and buy-in, and national advocacy (country readiness) .

### Conditions for scale-up

Conditions for scale up focuses on creating sustainable access conditions through regulatory approvals prior to market access.

#### Regulatory approvals

Regulation costs were deemed a significant barrier to market access, with many participants viewing the cost of application to the Therapeutic Goods Administration as prohibitive due to it being a listed fee and not proportionate to population size/market availability. Ultimately, this can create a significant fiscal barrier to market entry: *For someone to actually put a test up to TGA and get it approved*,* it’s big money* (Participant 16). Participants held the perception that the predicted return on investment is often insufficient to incentivise companies to apply for approvals to access smaller markets, such as Australia. As prevalence and testing needs vary by disease and setting, and some POC testing technologies may be limited to testing for a single disease, there were recommendations that application fees should be based on testing potential. That is, scale of market access should be considered for companies applying for regulatory approvals.

*From a registration perspective for these companies registering*,* they probably need some sort of guarantee on volumes or alternatively need to lower the barrier to registering a particular product in terms of those costs for them to recoup or*,* obviously*,* they need to sell so many devices or tests before they can recoup that. I think that’s the big barrier for them at the moment. (Participant #2)*

Regulatory rigor was also considered a key barrier to market access. It was recognised that the risk of the test (that is, the potential for level of harm of the disease being tested for, such as a false positive for HIV for example), should influence the extensiveness of regulatory rigour for in-country approval of diagnostics. One such consideration was the downgraded reclassification of some hepatitis C diagnostics in the USA which occurred during data collection.

*America […] have just regulated*,* downscaled the classification of hep C testing. […] What that’s done is they have acknowledged that because of its public health impact risk. […] The hurdles*,* the registration*,* they have been dropped so I have a feeling organisations such as [a diagnostic company] who aren’t registering a test in America*,* well now sort of sitting in the wings waiting for things to happen because now actually it makes more business sense to go in because the hurdles aren’t so high and there is a huge market there. So*,* I think there is opportunity for us to also think about why tests are ranked the way they are and what the requirements are to meet those*,* so in terms of their risks. So that’s just an example of somewhere else*,* where I think they’ve pragmatically gone “we don’t need to do that” […] and eventually it changes the landscape in America for access to tests. (Participant #26)*

### Establishing country readiness for scale-up

Establishing country readiness for scale-up encompasses securing political and financial support (national advocacy and funding) and ensuring programmatic and operational readiness (supportive policies, integration into national programs, and adequate health system capacity). Sustainable funding is a primary consideration of programmatic and operational readiness [21].

#### Funding models and affordable pricing (programmatic and operational readiness)

Several participants reflected on the long-term financial sustainability of POC testing as being integral to diagnostic companies’ decisions as to whether they invest in regulatory approval costs. It was viewed that without return-on-investment opportunities, many companies may otherwise view market access to be an exorbitant cost. Thus, here we consider funding models and affordable pricing as integral to pre-market considerations for scale up of POC testing, as scope may influence market access decisions. This process creates an additional barrier to market access for POC testing devices, but without which, it is anticipated the out-of-pocket costs would be an inhibitor to provider and patient uptake. Participant #15 highlights the political power dynamics at play and the fight for finite resources, whereby people – or groups – with the most influence can have greater clout rather than which devices have the most evidence behind them.

*The first, the biggest, and the most important enabler is the funding model. (Participant #1)*

*I do think that the decision of what goes on the Medicare reimbursable list and what does not requires lobbying and requires real engagement from probably the biggest influences in healthcare. (Participant #15)*

#### National advocacy: setting strong roadmaps for whole-of-country response

National strategies and international commitments were regarded as key policy enablers to scale-up. World Health Organization (WHO) guidelines inform health decision-making in most countries globally, with many countries signatories to WHO guidelines. It was viewed by participants that Australia’s national strategies for specific diseases are additionally useful as these strategies have already done the work of collecting evidence and developing advocacy for action.

*I think Australia having signed up to the WHO [hepatitis C virus] global targets is probably a system enabler. (Participant #8)*

*The capacity that the national [BBV and STI] strategies are very well put together and so it provides a really strong roadmap for which point-of-care testing could be implemented*,* because once you have those policy frameworks in place*,* that makes it a little bit easier to … for the government funders to then align funding and apply implementation efforts along the direction of the national strategies*,* so I think having a policy framework in place is really critical and Australia has done a really great job at having that policy framework. (Participant #22)*

Although participants noted that national strategies can foster scale-up, they also discussed the potential for misalignment or conflict between the recommendations of a strategy and what can be facilitated or achieved within current regulatory frameworks. Historically, diagnostic testing has typically not been conducted by primary care providers and instead managed by referral to a public or private company. Current regulatory frameworks reflect these traditional, centralised diagnostic pathways, which have very different mechanisms for managing diagnostic testing. POC testing is a diagnostic tool which can be decentralised and conducted in-house in the primary care setting. Thus, it has the potential to disrupt the current regulatory landscape. As the below participant describes, POC testing does not fit neatly into one regulatory domain, instead, straddling primary care *and* pathology.

*I think the biggest difficulty for the reimbursement of point-of-care testing is there is not really a precedent yet for us to build on*,* so the first thing is to be able to create that precedent*,* and point-of-care testing doesn’t really fit nicely into the existing Medicare Benefit Schedule where there is a strict pathology arm and a strict service arm*,* whereas point-of-care testing is a bit of both. So it creates a bit of a policy dilemma for [government] as to well*,* “where does this fit?”. (Participant #8)*

As noted in the quote below, such tensions in implementation systems, funding models and policy can hinder scale-up of POC testing technologies. Thus, it is not simply a matter of utilising existing policy to advocate for integration of POC testing technologies; there is also need for regulatory frameworks to adapt and create space for these disruptive technologies. A key difficulty highlighted by participants is identifying individuals or groups to champion and shepherd POC testing through the multiple mechanisms required for government approval and access to market.

*I think obviously someone needs to take on the regulatory and policy-based push and that’s kind of hard to pick. […] It needs to be some sort of invested yet widescale party that is willing to drive through things like the TGA [Therapeutic Goods Administration] regulatory stuff and the MSAC [Medical Services Advisory Committee] funding stuff at sort of a sustained level over time. I don’t really know who that would be. [laughs] I don’t think we really have anyone. (Participant #30)*

#### Advocacy: who is responsible for pushing the agenda?

Advocacy was widely viewed as reliant on key stakeholders, whereby pre-existing stakeholder relationships coupled with policy (or other) momentum acted as an enabler for scale-up readiness. However, in Australia, there is no body or group specifically tasked with advocacy to create market access for POC testing - i.e., provision of support to overcome regulatory (and other policy, political, economic, and social) hurdles. Thus, advocacy work often falls to peak bodies (an Australian term referring to national and state-level nongovernment bodies, typically operating as an umbrella group for smaller organisations, such as state or regional, and often tasked with advocacy responsibilities for the group or disease of interest). Yet, peak bodies may not always have capacity, resources, or influence to push this agenda. Several participants considered this a gap, and raised the question of: who takes responsibility for pushing the agenda?

*Who should be doing this? Someone should be doing this but it kind of falls between the cracks. (Participant #12)*

Related to questions of responsibility, participants raised concerns regarding risk of advocacy and policy burnout with some directly commenting on “*policy fatigue and policy desensitisation*” (Participant #21) as inhibiting country preparedness. The momentum of research, demonstrated through production of evidence, and successes sustained through implementing evidence into practice, were viewed as enablers. Yet, participants cautioned that such momentum can have a flip side, drawing attention to the need to address the issue of fatigue within advocacy in ensuring policy change efforts.

*“This is vitally needed public health technology”*,* but our advocacy muscle as a public health community is pretty weak and I think we are not doing our job properly. (Participant #12)*

Ultimately, country readiness to enable scale up requires alignment and cohesion of overlapping systems, including stakeholder agreement. As the below participant discusses, enabling scale up cannot be achieved when systems work in isolation.

*The starting premise of point-of-care is that it actually requires a whole range of systems and stakeholders to kind of talk to each other. […] I think right now is a really nice example of some really good leadership coming out from different spaces that really want to work together and these kind of aspects of leadership being like*,* “let’s do this”*,* and then getting people behind it and really working for the broader purpose of the community*,* I think that is like a really critical ingredient of success here. […] a bunch of things that have come together*,* but it’s not seen as an isolated activity. (Participant #19)*

Identification of stakeholders who might not be part of collaborative professional advocacy but who may have different priorities and concerns was also recognised. Participants perceived that pathology stakeholders may act as a barrier to scale-up of POC testing, given their market stronghold and existing infrastructure under universal healthcare. Specifically, it was suggested that the pathology industry has political clout and may be less inclined to support policies which would reduce their revenue, such as diversion of testing from pathology laboratories to management of diagnostic processes within primary care settings.

*The other policy dilemma […] is of course the pathologists don’t want [government] to remove their income from the [Medicare Benefits Schedule]*,* so there is a sensitivity I guess there as well. (Participant #8)*

*I think the biggest roadblock is the funding issue and so and I think that is interconnected with the fact that right now laboratories would be set to lose quite a bit of income. (Participant #15)*

#### COVID-19 POC testing: case example of system readiness

COVID-19 POC testing was described as a powerful case example to draw on when advocating for infectious diseases POC testing. The pandemic very quickly forced recognition that POC testing *can* aid in triaging patients through swift diagnosis. Although many of the regulatory and other health systems create barriers to enable country readiness, rapid implementation of COVID-19 POC testing has demonstrated an environment ripe for enabling scale-up (e.g., the infrastructure is achievable with government will and financial support). Participants suggested that the successful scale up of COVID-19 POC testing provides a launching platform for advocacy efforts for other infectious diseases POC testing.

*COVID has offered a new opportunity for the potential expansion of the use of point-of-care tests for infectious diseases that government has recognised an important need for being able to have tests that people can do at the point-of-care or at near the point-of-care […]. And I think that that has generated momentum in terms of being able to get political commitment to figure out a mechanism by how do we sort out where the point-of-care tests fit. (Participant #22)*

## Discussion

Drawing on interviews with 30 stakeholder participants with expertise in infectious diseases and/or POC testing identified several health systems enablers and barriers to national scale-up of POC testing. Existing regulatory frameworks were regarded as a key barrier to creating conditions for scale up, while national strategies were regarded as an important enabler to country readiness. Financial sustainability of POC testing in the primary care setting was deemed a complex hurdle, requiring advocacy to drive political will and overcome regulatory barriers. However, responsibility for advocacy remained undefined, with many participants viewing this as a critical barrier to enabling country readiness. COVID-19 was regarded as a case example demonstrating that market access barriers for POC testing can be swiftly addressed with government support.

Regulatory frameworks have been recognised as a critical consideration of large scale-up initiatives, with regulatory approvals deemed an important component of ensuring sustainable access conditions [21]. Participants denoted the regulatory divide where POC technologies do not fit within current regulatory frameworks. Stringent regulatory rigour was deemed as necessary to ensuring high quality technologies, but many participants perceived the limited return-on-investment opportunities amid Australia’s smaller population made the costs of application a substantial barrier. By way of comparison, in the United States, the Point of Care Technology Research Network was created to facilitate a “pipeline of point-of-care technologies with commercialization potential” that “enables incorporation of clinical and user needs”, as well as providing expertise to “address barriers to commercialization and implementation” [23]. Such an organisation enables pre-screening of potential diagnostics prior to application enabling a smoother pathway from design to market for POC technologies. In response to the changing landscape of diagnostics, including where, how, and by whom tests are conducted, the FDA has introduced a Waiver System, whereby new in vitro diagnostics (such as POC tests) are assessed using a “criteria scorecard” [24]. The seven categorisations within the scorecard assess (1) knowledge required to operate the test, (2) training and experience required for analysis phases, (3) reagents and materials preparation, (4) characteristics of operational steps, including their complexities, (5) calibration, quality control, and proficiency, testing materials, 6) test system troubleshooting and equipment maintenance, and 7) interpretation and judgment [24]. Such a model enables tests to be rapidly assessed (within two weeks) against the seven criteria, thereby ensuring timely processes along the regulatory approval pipeline. Australia’s TGA approval system would benefit from recognising the smaller market for POC diagnostics, and bespoke assessment processes for POC tests like the US’s Waiver System. The “Strengthening diagnostics capacity” resolution (WHA76.5) was adopted at the 76th World Health Assembly, calling upon member states “to consider, as appropriate, legislative, administrative or policy measures to prevent anti-competitive practices that hinder access to diagnostics” (Item 11) [25]. As Australia is a member state, overcoming existing regulatory hurdles is imperative to meet the Strengthening diagnostics capacity resolution.

Participants widely viewed Medicare reimbursements (Australia’s universal healthcare scheme) as the most appropriate sustainable funding model as part of establishing country readiness. For a new technology to be reimbursable under the Medicare Benefits Schedule, this requires an extensive application and additional fees, thereby adding to the already exhaustive regulatory processes. To the authors’ knowledge, only one application for POC infectious diseases testing for use in the primary care setting has been submitted to the Medical Services Advisory Committee (the committee tasked with approving public health interventions for Medicare reimbursement) (See: [26]). This suggests that, while participants’ views that Medicare reimbursement is an important component of country readiness and financial sustainability of POC testing, the feasibility of submitting POC tests for reimbursement consideration and approval is a significant hurdle. A government-led inquiry into the “approval processes for new drugs and novel medical technologies in Australia” made several recommendations to better enable market access for novel technologies, such as POC testing, including ensuring appropriate expertise on the Medical Services Advisory Committee relevant to the diagnostic being reviewed and timelier assessment processes (Recommendation 15) [27]. Decisions of health technology assessments, such as applications to the Medical Services Advisory Committee, are largely based on cost-effectiveness [28]. Given that point-of-care diagnostics cost more per test than laboratory-based testing, there may be an economic barrier to Medical Services Advisory Committee approvals which ultimately privileges pathology providers for their lower cost per test services. Considerations of setting (e.g., remoteness to pathology), populations (e.g., transient and those who may be infrequently engaged in health care services, as well as populations at higher risk of exposure), should factor into cost-effectiveness analyses, whereby POC testing which leads to timely treatment (and prevention of ongoing transmission) may be more cost-effective than standard testing approaches whereby infection may go undiagnosed (or delayed diagnosis) via standard testing pathways [29].

COVID-19 responses have instigated legislative changes to regulatory frameworks and environments in countries around the world in order to fast-track access to public health tools [30]. However, while these legislative changes have been enacted during the pandemic, many countries implemented sunset clauses for such modifications to automatically expire or be considered for extension [30]. The example within our data regarding rapid scale-up of COVID-19 POC testing demonstrates that regulatory and other hurdles can be overcome swiftly. Rapid government response implemented with clauses to limit future progress relating to POC testing is perhaps indicative of motivation. That is, an outbreak of highly infectious disease can set the conditions necessary for the types of national advocacy essential for scale up of POC testing. Participants suggested that rapid scale-up and implementation of COVID POC testing could be utilised as an advocacy tool when considering POC testing for other infectious diseases. However, what is not discussed in the data is whether the type of disease (i.e., one that is highly stigmatised such as hepatitis C vs. something more common) and the population affected (e.g., people who inject drugs vs. the general population being at risk) influences political will to mobilise change and expend political capital.

The role of national strategies to provide leverage for advocacy efforts in establishing country readiness was regarded as an essential piece of the advocacy toolkit, though misalignment between national strategies and regulatory market access were seen as impeding such efforts. During data collection, the United States reclassified some hepatitis C diagnostic tests to a lower grading, enabling manufacturers of these tests to seek marketing clearance via a less “stringent” Food & Drug Administration review pathway (19 Nov 2021) [31]. Importantly, this reclassification was identified as “benefit[ing] the Department of Health and Human Services’ Viral Hepatitis National Strategic Plan” [31]. Together, this regulatory and policy response evidence the disconnect that can occur between policy (such as national strategies) and regulatory frameworks, and that policy modifications can be implemented to better align public health policies with the regulatory environments within which they operate.

This study has limitations. While efforts were made to ensure broad representation of expertise and policy experience across infectious diseases and health systems, it was not possible to interview all relevant stakeholders. However, participants provide insights across a range of scale-up considerations with ample depth provided within the data. Additionally, the Unitaid scalability framework is designed for Unitaid grant applicants and implementers, with some elements of the framework beyond the scope of this analysis (e.g., components of the framework relevant to scale-up models enacted through Unitaid funding, including transitions and commitments for scale-up) [21].

## Conclusions

This qualitative analysis identified key pre-market barriers to creating conditions and enabling country readiness for infectious disease POC testing in primary care across Australia. Drawing on interviews with key experts nationally, this study identifies the ways in which existing regulatory frameworks inhibit market access and impede sustainable funding mechanisms. Participants noted the ambiguity of national policies which, on paper, foster implementation of POC testing, whilst broader regulatory models impede their market entry. National advocacy was identified as critical to push forward the POC agenda and gain government support in efforts to improve access to timely diagnostics for all Australians. Future research should explore how other countries are addressing the issues raised in this analysis, including government, practitioner and civil society perspectives, and provide recommendations which could be adopted by countries considering national-level scale up of POC testing.

## Supplementary Information

Supplementary Material 1.

## Data Availability

Analysis draws on transcripts with 30 interview participants. Data has not been made publicly available to ensure confidentiality of participants.
