# Supplementary material for: Pre-market health systems barriers and enablers to infectious diseases point-of-care diagnostics in Australia: qualitative interviews with key informants
Source: BMC Infect Dis. 2024 Nov 19;24:1317. doi: 10.1186/s12879-024-10214-5 (PMC11575463; doi:10.1186/s12879-024-10214-5)
Supplement: Supplementary file 1 — Supplementary Material 1. [file 12879_2024_10214_MOESM1_ESM.docx]

**Interview Guide: Integrated point-of-care testing for infectious diseases in primary care settings**

Gender:

Location (state/territory):

Section One: Professional and organisational background and experience

1. Can you tell me a bit about your current role, and your organisation? (*Prompt: Length of time in current role; professional background and training)*
2. Can you tell me a bit about your/your organisation’s experience with point-of-care (POC) testing/treatment? *(Prompt: Have you (or your organisation) had any previous involvement with POC technology? Prompt: Populations, tests, settings you/your organisation have been involved with? Prompt: Views about the value/costs of POC testing)*

Section Two: Scale up of existing POC tests

1. What are some of the priorities for POC tests? Why? (*e.g., which specific infections get prioritised in different times and communities; and why? What are some of the other priorities for POC tests in remote communities? Prompt: Views on competition for attention of different infections*)
2. What does POC testing/POC scale-up mean to you?

1. What evidence do you think is relied upon make decisions about scale-up? Is there any other evidence that is important but that isn’t currently used in these decisions? (e.g., community knowledge/patient preferences and expectations)
2. From your perspective, what are some of the key things to think about when making decisions about scale-up for POC testing? *(e.g., governance/accreditation, quality, testing and record management, staff training, setting, costs, infrastructure, community consultation/acceptability, sustainability)*
3. Could you talk me through how you would approach the scale-up of POCT? (*Prompt:* *What would be your strategy? How would your strategy change for different populations/settings? Prompt: Under what circumstances would you want to scale-up? (e.g., what settings, population groups, outbreaks/epidemics)*
4. When you are making decisions about scale-up, what are some of the critical moments in decisions/planning?
5. Who are the key actors involved in decisions about scale up? (*Prompt: Different agendas/priorities*)
6. What would be your concerns about scaling up? What about some of the markers of success that you would use to judge successful scale up? (*Prompt: Can you give me any examples of previous successes/failures?*)
7. What might be some of the enablers/facilitators at the health system level of scale-up plans/ integration into service delivery? (*Prompt: How important is adaptability/flexibility?*)
8. What about some of the roadblocks/challenges of scaling up of POC testing/integration into service delivery? (*Prompt: Where are they? What might take you off track?* (*e.g., workforce availability, provision/cost of infrastructure, setting*))
9. What is the way forward for planning for scale-up for POC testing? What needs to happen to ensure success? (*Prompt: What strategies would you recommend for ensuring optimal integration of POC testing into primary care delivery?*)
10. At a systems level, COVID-19 shows what can be done in POC testing scale-up. What has COVID-19 POC testing taught us about POCT scale up decisions and planning? (*Prompt: How might POCT decision-making/planning be transformed as a result?*)

Section Three: Opportunities for POC testing scale up in the future

1. Thinking about future opportunities for new POC tests. What other infectious diseases could POC testing be used for? How do you decide what new tests should be prioritised for scale up? (*Prompt: What parameters do you need to think about?* *e.g., screening for infection versus syndromic management/reducing inappropriate antibiotic tx, prevalence of infection, ability to change clinical management, faster treatment, saving staff time*)
2. What other infectious diseases do you think would benefit most from POC testing in remote areas (e.g., respiratory infections, hepatitis B/C, impetigo (caused by group A strep/staph), pharyngitis (caused by group A strep), gastro (bacterial, viral, parasitic pathogens).
3. Should POC be considered in urban areas where labs are close by? If yes, what parameters should be considered? (e.g., high loss to follow up of patients, homelessness).
4. What funding models should be considered to sustain infectious disease POC testing?
5. Is there anything else that we haven’t discussed yet that is important to share about your experiences or views?
